# Crown and Bridge Work

**Published:** 1920-09

**Authors:** F. W. Frahm

**Affiliations:** Los Angeles


### CROWN AND BRIDGE WORK

BY F. W. FRAHM, A.B., PH.G., D.D.S., LOS ANGELES

Let us consider the earmarks of removable bridge work and partial denture work. Is there a man who can draw a line of demarkation between these two systems of restorative dentistry? Is it possible to develop a technic or system for the one that does not apply to a large extent to the other? Would the principles involved in the application for the one have no bearings upon the other? Is a clasp-anchored partial denture of gold construction a removable bridge, or is it just an ordinary partial denture? Would not such a partial denture fulfill many of the features commonly attributed to that distinctive class known as "removable work"?

What distinctive features should every piece of removable bridge work possess?

1. It must be designed, constructed and adjusted in a manner that will not offend the remaining units of mastication or their associated physiological functions.

2. It must fulfill to the greatest possible degree the esthetic requirements of each case and so harmonize with its surroundings that it will not attract undue attention unto itself.

3. It must be constructed in the simplest and strongest manner to insure a maximum service to the patient at a minimum inconvenience.

4. The bridge itself should function with its opposing masticating units without any unusual wear or strain upon such units. This would necessitate the normal interlocking of cusps and the apposition of both the active and passive inclined planes in as perfect a manner as tooth positions and the movements of the mandible will permit.

5. The foundation work of such appliances should be extended into areas that will offer as much resistance against direct and indirect stress as it is possible to obtain. The inclined planes both active and passive should be posed in a manner that will admit of the free movement and function of the mandible without any unusual strain upon the cusps of the several units of the bridge.

6. The several units of the bridge and their surrounding parts should resemble as closely as possible the lost organs or units. The frictional appliances, either simple or complex, should be placed in as secluded a position, consistent with their function, as is possible for the case.

7. The detailed construction of the bridge and its retainers, either internal or surface, should offer the least amount of difficulties toward keeping either or both in a hygienic condition.

8. The appliance or bridge should not provoke certain neurotic manifestations or conditions, or aggravate those with which the patient is already afflicted. Our present modes of living and the pursuits we must engage in to provide that living are producing an ever-increasing number of neuresthenics and much increase of nervous people. Any appliance in the mouth of such people that would keep them conscious of its presence and aggravate the nervous affliction is contra-indicated. This past week a young woman who is head of a department in one of our largest department stores, made the following demand: "Doctor, I must have those two spaces filled, but, whatever is done, they must not move to such a degree that I will be conscious of it. Such a bridge would drive me crazy." It is a sad commentary on life, I am sure, but the demands upon the nervous energy of our nation are here and we, as dentists, must do as little along the path of aggravation as it is possible if we hope to claim success for this branch of our work.

These few brief remarks concerning some of the features every removable appliance should have, can also be applied to partial denture work. Either class of appliances serves the same functions. Theoretically, the one may be more desirable from either operator or patient's standpoint, but, practically, there is very little real difference. Both should provide against the early disintegration and loosening of the abutment and supporting teeth. They should restore the masticating apparatus and its associated parts to as near normal function as possible, without unusual pressure upon the ridge under the saddle.

*The Foundation.* Every bridge of the removable class has a double foundation; namely, the ridge and the internal parts of the anchors. The former is usually of a yielding character and more or less undependable. The internal parts of the anchors are a part of one or more of the associated teeth which do not move and function with the yielding tissue that covers the ridge. The teeth move in function as single units and any appliance that calls for the support of two or more teeth must be constructed in a manner that will limit to the least degree such individual movements. It may be well to remember that certain teeth on one side of each arch have movements common to both sides, and the partial binding or limiting of such movements is not to be regarded harmful or destructive. The mesio-distal movement of any of the molars or bicuspids is negligible, because the natural contact of each tooth in the arch limits such a movement. The rotary or torsion movement of these teeth is of little value as far as function is concerned. The health of the tissues surrounding a tooth and the peridental membrane does not depend upon these two movements of the tooth. A careful study of the facet worn upon the approximal surface of a molar or bicuspid will quickly convince the observer that it is perfectly flat in every direction. If this rotary movement existed to any degree, it would manifest itself by giving us a convex facet upon these surfaces of the tooth at the point of contact. The remaining movements that a molar or bicuspid may enjoy during function are not restricted by our present types of removable bridges or partial dentures, hence the wail about the limitation of tooth functional movements need not concern us in the least.

The two forms of retention now largely used to anchor our bridge work depends upon either the internal or external grip. Those attachments that are confined within the bounds of a crown, Carmichael, Kennedy, or inlay, are classed as those of the internal retention, while the stop-clasp, the crib and kindred appliances are known as the external anchor. Any of these will admit of such movements as the tooth itself may allow. If there is more play than the detail of construction permits, there will be an injury done to the tooth carrying such an anchor, thus *impairing the bridge foundation to that degree.*

The bony ridge and its overlying area of mucous and sub-mucous tissue is always the questionable element in our removable bridges that depend upon saddles for a part of their support. The underlying osseous structures that are usually involved are those that gave support to the missing teeth; namely, the alveolar crest of the maxilla or mandible. When such teeth have been removed, the alveolus is resorbed to a large extent, if not entirely. Such resorption after extraction is entirely physiological in its nature. When resorption follows the progress of diseases or injury, it is both physiological and pathological, however, the results are similar, but more extensive. This resorption from either cause may be hastened, by the application of gentle pressure in the presence of chemical and thermal irritation. However, some cases do not even need this gentle pressure to remove the alveolus, the resorption taking place in spite of everything we can do.

It is also a recognized scientific fact that the osseous structure made up of the Haversian system will resorb under either constant or intermittent gentle pressure. A very common and quite general manifestation of this truth may be had from the people who wear the "Shure-on" nose-grip eye-glasses. The writer wore such glasses for four or five years and then laid them aside, but the two depressions in the nasal bones are still there, and, no doubt, will remain there, for he has not yet developed the technic of scientific stimulation of some of the internal secretory glands that influence the deposit of bone in selected areas. If he did know, he would also want to know the exact method of retarding or checking such deposits in order to prevent the growth of an abnormal nose, or what not. The Haversian system supporting the alveolus is no different from that found in other osseous parts of our bodies. A spot saddle or even larger saddles will imbed themselves in the ridges on which they rest, unless the teeth that also enter into the support of the bridge carry a major portion of the load. It is a recognized fact that most of our removable bridges that have only saddle support, and our partial dentures, rest upon a questionable, unreliable and changeable foundation. Provisions that will care for these changes and correct the evil tendencies should be made at the time that the appliances are constructed and placed.

Knowing that the teeth which are intimately associated with our appliance will have to assume the major part of the load, we must give them a careful examination to determine the amount of additional work they may perform. Any tooth that has had, or is at the present time suffering from periodontoclasia, should never be considered a fit member of the bridge foundation. The skiagraph may show very little or no radiolucent areas, thus encouraging the operator to use the tooth or teeth for a part of his foundation. The writer is convinced that such a procedure is certainly inviting trouble, if not a positive step toward malpractice. The use of pulpless teeth for a part of our foundation will be admissible, providing the canal has been filled and there is no evidence, clinically or radiographically, of trouble at the apical area, and that such a tooth will not be overburdened with the new load that is to be placed upon it.

The teeth that are suffering from a lateral abscess or other pathological conditions should also be very carefully considered before any use of them is planned. A clinical and radiographic study of the entire foundation should be made before plans for any appliance can even be considered. The next step in the work is a careful study of the amount and direction of stress the appliance will be subjected to. This will be our next study.—*Pacific Dental Gazette.*

